# N^ϵ^‐Carboxymethyllysine Increases the Expression of miR‐103/143 and Enhances Lipid Accumulation in 3T3‐L1 Cells

**DOI:** 10.1002/jcb.25576

**Published:** 2016-06-06

**Authors:** Ann‐Katrin Holik, Barbara Lieder, Nicole Kretschy, Mark M. Somoza, Sandra Held, Veronika Somoza

**Affiliations:** ^1^ Faculty of ChemistryDepartment of Nutritional and Physiological ChemistryUniversity of ViennaAlthanstraße 14Vienna 1090Austria; ^2^ Faculty of ChemistryChristian Doppler Laboratory for Bioactive Aroma CompoundsUniversity of ViennaAlthanstraße 14Vienna 1090Austria; ^3^ Faculty of ChemistryDepartment of Inorganic ChemistryUniversity of ViennaAlthanstraße 14Vienna 1090Austria; ^4^ Department of Food ScienceUniversity of WisconsinMadisonWisconsin

**Keywords:** N‐epsilon‐carboxymethyllysine, ADIPOGENESIS, 3T3‐L1, microRNA

## Abstract

Advanced glycation endproducts, formed in vivo, but also by the Maillard reaction upon thermal treatment of foods, have been associated with the progression of pathological conditions such as diabetes mellitus. In addition to the accumulation with age, exogenous AGEs are introduced into the circulation from dietary sources. In this study, we investigated the effects of addition of free N^ϵ^‐carboxymethyllysine (CML), a well‐characterized product of the Maillard reaction, on adipogenesis in 3T3‐L1 preadipocytes. Treatment with 5, 50, or 500 μM CML resulted in increased lipid accumulation to similar extents, by 11.5 ± 12.6%, 12.9 ± 8.6%, and 12.8 ± 8.5%, respectively. Long‐term treatment with 500 μM CML during adipogenesis resulted in increases in miR‐103 and miR‐143 levels, two miRNAs described to be involved in impaired glucose homeostasis and increased lipid accumulation. Furthermore, the expression of genes associated with these miRNAs, consisting of *Akt1*, *PI3k*, and *Cav1* was regulated by CML. Short‐term treatment of mature 3T3‐L1 adipocytes with CML resulted in decreased basal glucose uptake. These results, indicate that the addition of protein‐free CML to 3T3‐L1 cells influence parameters associated with adipogenesis and glucose homeostasis at transcriptional, and functional level; this indicates that free CML derived from exogenous sources, in addition to protein‐bound CML may be relevant in this context. J. Cell. Biochem. 117: 2413–2422, 2016. © 2016 The Authors. *Journal of Cellular Biochemistry* Published by Wiley Periodicals, Inc.

**Abbreviations**
AGE: advanced glycation endproduct
BSA: bovine serum albumin
CML: N^ϵ^‐carboxymethyllysine
MTT: 3‐(4, 5‐dimethylthiazolyl‐2)‐2, 5‐diphenyltetrazolium bromide
PBS: phosphate buffered saline
RAGE: receptor for advanced glycation endproducts

Advanced glycation endproducts (AGEs) comprise a highly diverse class of molecules formed by the Maillard reaction between amino groups and reducing sugars upon thermal treatment of foods, and in vivo, by long‐term interaction of proteins with glucose, particularly under hyperglycemic conditions [Makita et al., 1992; Jousse et al., 2002]. Exogenous AGEs, typically encountered in a Western diet, are at least partially absorbed into circulation where they add to the endogenous AGE level [Uribarri et al., 2005; Somoza et al., 2006; Sebekova et al., 2008; Birlouez‐Aragon et al., 2010]. Among the group of AGEs, N‐ϵ‐carboxymethyl‐l‐lysine (CML), a well‐characterized, stable [Ahmed et al., 1986; Kislinger et al., 1999; Uribarri et al., 2010] member, and major epitope of AGE‐modified proteins [Ikeda et al., 1996], has been detected in a wide range of foods, its concentration reaching up to 84.8 mg per average portion size in meat dishes [Hull et al., 2012]. In a study on the dose‐dependent utilization of CML in rats, 30% of dietary CML has been shown to enter the blood stream [Somoza et al., 2006]. Furthermore, an increase in AGE plasma concentrations by 65% after 2 weeks of consuming a diet high in AGEs has been demonstrated in diabetes type 2 patients, while a decrease of 30% was observed in a group served meals with only one‐fifth of the AGE load [Vlassara et al., 2002]. Similarly, an increase in CML plasma concentrations by 46% was detected in infants fed exclusively infant formulas, high in CML due to heat treatment, compared to breast‐fed infants. In addition, a 60‐fold increase in the urinary excretion of CML was observed, indicating rapid excretion of absorbed CML via urine [Sebekova et al., 2008].

An increase in AGE levels originating from exogenous sources may be of particular importance in compromised individuals, already exhibiting elevated plasma concentrations, for instance, diabetics with impaired renal function [Lieuw et al., 2004]. Additionally, higher local AGE levels may be reached independently of pathological conditions due to accumulation in tissues. Gaens et al. [2014] reported a receptor for advanced glycation end products (RAGE) mediated trapping and accumulation of circulating protein‐bound CML in adipose tissue, which decreases plasma CML levels in obese individuals. In addition, plasma CML levels were shown to be strongly associated with obesity‐related insulin resistance [Gaens et al., 2014]. Similarly, Cai et al. [2012] showed that exogenous AGEs promote insulin resistance and diabetes in C57BL6 mice. In their study, four generations of C57BL6 mice were fed with synthetic methylglyoxal derivatives or an isocaloric control chow. Between F0 and the following generations, F1–F3, gradual increases in CML serum concentrations were observed. Furthermore, mice of the F3/methylglyoxal group showed premature insulin resistance as well as increased adiposity [Cai et al., 2012]. In addition to in vivo effects, several studies on the influence of AGEs on adipogenesis have been conducted using cell line models of adipogenesis. Chen et al. [2012] described an AGE‐RAGE‐mediated induction of adipogenesis in senescent preadipocytes by inhibition of p53 in murine cells. A study by Jia et al. [2012], using methylglyoxal reported an up‐regulation of the Akt1 pathway, thereby, stimulating adipogenesis in murine 3T3‐L1 cells. Moreover, using the model system of obese Zucker rats, the authors showed increased methylglyoxal accumulation in the adipose tissue of the animals [Jia et al., 2012]. Gaens et al. [2014] observed a strong increase in CML levels during the differentiation of human SGBS preadipocytes. The effects of AGEs on adipogenesis may be of particular interest in the context of adipogenesis in adults, where one‐tenth of the adipocytes are renewed every year [Hausman et al., 2001; Spalding et al., 2008]. Furthermore, diet‐induced hyperplastic, nonhypertrophic obesity has been shown in experiments on rats on highly palatable chow, during which adipocyte number increased, and remained high after a return to a standard diet, while only adipocyte size returned to normal [Faust et al., 1978]. Adipose tissue is not only important in the context of lipid storage, adipokines secreted from adipose tissue have been shown to be involved in metabolic homeostasis by interacting with peripheral, or central organs [Harwood, 2012]. CML‐RAGE interactions have been demonstrated to be involved in the dysregulation of adipokines, and hence, aid obesity‐related insulin resistance [Gaens et al., 2014].

As several studies show the influence of diverse AGEs on adipogenesis in adult organisms via adipocyte renewal and hyperplasia, we investigated the effects of free CML on adipogenesis in 3T3‐L1 cells, a commonly used adipogenesis model cell line, by analyzing lipid accumulation, and gene expression of key genes involved in adipogenesis such as *PPARγ* and *C/EBPα* [Farmer, 2006]. Since, microRNAs have been linked to the regulation of adipogenesis [McGregor and Choi, 2011], we performed measurements on the miRNA profile of 3T3‐L1 preadipocytes using custom miRNA microarrays and digital droplet PCR. Furthermore, we determined whether CML exerts influence on fully differentiated adipocytes, which may aid in the development of adipocyte hypertrophy or modify adipocyte properties, in terms of gene expression, free fatty acid, and glucose uptake.

## MATERIALS AND METHODS

Chemically synthesized, protein‐ and hydrochloride‐free N‐ϵ‐carboxymethyl‐l‐lysine was obtained from Iris Biotech (Marktredwitz, Germany). All other reagents were purchased from Sigma–Aldrich (Austria) unless stated otherwise.

### CELL CULTURE

The murine fibroblast cell line 3T3‐L1 was purchased from the American Type Culture Collection. Preadipocytes were maintained in growth medium consisting of Dulbecco's Modified Eagle Medium (4.5 g L^−1^ glucose) supplemented with 10% fetal bovine serum, 4% l‐glutamine (8 mM), and 1% penicillin/streptomycin (corresponding to 100 units penicillin and 171 μmol streptomycin L^−1^) at 37°C and 5% CO_2_ in a humidified incubator. Differentiation into adipocytes was induced as reported by Riedel et al. [2012]: 2 days post‐confluence, 3T3‐L1 cells were treated with 3‐isobutyl‐1‐methylxanthine (0.5 mM), dexamethasone (1 μM), and insulin (10 μg mL^−1^) supplemented growth medium for 48 h. The cells were then maintained in maturation medium (growth medium containing 10 μg mL^−1^ insulin) for 48 h and in growth medium for another 5 days prior to experiments unless stated otherwise.

### CELL VIABILITY ASSAY

In order to exclude negative effects of CML on cell viability, MTT (3‐(4, 5‐dimethylthiazolyl‐2)‐2, 5‐diphenyltetrazolium bromide) assays were performed. 3T3‐L1 were seeded into 96‐well plates and differentiated as described above but with the modification of treatment with maturation medium for 10 days. CML in concentrations ranging from 0.5–500 μM was added with the corresponding medium starting at either differentiation or maturation. On day 12 after induction of differentiation, the medium was exchanged with 100 μL of MTT solution (1 mg mL^−1^ MTT reagent dissolved in serum‐free growth medium). The MTT solution was aspirated after the formation of purple crystals was observed (∼15 min). The formed formazan precipitate was dissolved in DMSO (150 μL) and the absorption recorded using a Tecan infinite M200 plate reader (Menningen, Switzerland). In order to exclude negative effects of the test compound on cellular metabolic activity, only treatments reaching values of at least 90% in relation to untreated controls were used in further experiments.

### OIL RED O STAINING

Oil Red O lipid accumulation was assessed as described previously by Riedel et al. [2012]. Briefly, 3T3‐L1 preadipocytes were seeded into 24‐well plates at a density of 1.0 × 10^4^ cells cm^−2^. Differentiation and treatment with test compounds was carried out as described above in the cell viability assay (0.5–500 μM CML). Cells were fixed by incubation with 10% (v/v) formaldehyde in phosphate buffered saline (PBS, pH = 7.4) for 1 h prior to treatment with Oil Red O staining solution (200 μL, 21 mg mL^−1^ Oil Red O reagent in 60% (v/v) isopropanol) for 10 min. After removing dye excess by washing four times with distilled water, Oil Red O inside lipid droplets was dissolved in isopropanol (750 μL). The absorption of the dissolved dye was recorded at 520 nm using the Tecan infinite M200 plate reader.

### miRNA ANALYSIS

#### miRNA microarrays

MicroRNA micorarrays were synthesized using maskless microarray synthesis as described by Agbavwe et al. [2011], with a modified reaction chamber allowing for the simultaneous synthesis of microarrays on two independent glass substrates as described by Sack et al. [2013]. The customized microarray comprises probes for all known mature mouse miRNA sequences from release 19 of miRBase [Griffiths‐Jones et al., 2006, 2008; Kozomara and Griffiths‐Jones, 2011], as described previously by Rohm et al. [2015]. For miRNA experiments, cells were differentiated using the standard procedure described above. CML in a concentration of 500 μM was added throughout the differentiation process, starting 2 days post‐confluence. Fresh CML was added every second day with the corresponding medium. On day 9, after induction of differentiation cells were lysed and total RNA isolated using the RNeasy Lipid Tissue Kit (Qiagen, Hilden, Germany). For preservation of miRNAs during washing RW1 buffer was exchanged with RWT buffer. The concentration and quality of the isolated RNA was analysed using the Tecan infinite M200 plate reader and the determined concentration used for normalizing the RNA input in down‐stream steps. The labelling reaction was based on a method by Wang et al. [2007]. A total of 300 ng RNA was used in the labelling reaction, consisting of synthetic spike‐in controls, phosphate‐cytidyl‐uridyl‐DY547‐3′ RNA dinucleotides (50 μM, Thermo Fisher Scientific), ATP (1 mM), Tris‐HCl (pH 7.8, 50 mM), MgCl_2_ (10 mM), DTT (1 mM), bovine serum albumin (BSA, 10 μg mL^−1^), DMSO (25% (v/v)), and T4 ligase (20 U, New England Biolabs). The reaction was incubated in the dark at 4°C for 2 h prior to isolation of the labelled miRNA using MicroBioSpin 6 columns (Bio‐Rad). The purified miRNA was used in a hybridization reaction consisting of Tween20 (0.01% v/v), BSA (0.06% w/v), EDTA (20 mM), Na^+^ (1 M), and MES (100 mM). Each microarray was hybridized with two independent samples in duplicates using customized SecureSeal adhesive hybridization chambers (Grace Biolabs). Samples were hybridized for 24 h at 42°C under constant rotation, washed, and scanned using a GenePix 4400A microarray scanner (Molecular Devices, Sunnyvale, CA). The scanned images were analyzed using NimbleScan 2.1 software (NimbleGen, Madison).

#### miRNA digital droplet PCR

Similarly, as described in the microarray section, miRNA was isolated from mature adipocytes after treatment with CML (500 μM) for 9 days, untreated control cells, and undifferentiated 3T3‐L1 fibroblasts. The extracted miRNA was reverse transcribed using the TaqMan miRNA Reverse Transcription Kit (Life Technologies) with specific primers for mmu‐miR‐143‐3p, mmu‐miR‐103‐1‐5p, and the let‐7 group (Life Technologies). The generated cDNA samples were added to PCR reaction mix consisting of PCR droplet supermix (Bio‐Rad) and specific TaqMan probes for the targeted miRNA (Life Technologies). After partitioning the prepared sample mix into 20,000 single droplets using a droplet generator (Bio‐Rad), the PCR amplification was carried out in a C1000 Touch Thermal Cycler (Bio‐Rad). The amplified samples were transferred into the QX200 Droplet Reader (Bio‐Rad) in which approximately 15,000 droplets were counted for every sample. The absolute concentration (copies μL^−1^) was calculated using QuantaSoft (Bio‐Rad).

### qRT‐PCR

Fully differentiated 3T3‐L1 cells were incubated with CML (500 μM) for 30, 60, or 90 min prior to RNA isolation using the RNeasy Lipid Tissue Kit (Qiagen, Hilden, Germany). In addition, RNA from untreated mature adipocytes and cells, which had been treated with CML (500 μM) for 9 days, was isolated. A total of 2 μg RNA of every sample was used in the reverse transcription (high capacity cDNA Kit, Life Technologies). Fast SYBR green master mix (Life Technologies) was used in qRT‐PCR experiments and the reaction monitored in a StepOnePlus (Applied Biosystems) device. The sequences of the reverse and forward primers are shown in Table I. Primers were designed with NCBI Primer Blast or taken from PrimerBank [Wang and Seed, 2003; Spandidos et al., 2008, 2010]. Hypoxanthine guanine phosphoribosyl transferase (*Hprt1*) was chosen as endogenous control [Han et al., 2002]. The concentration of input target mRNA (N0) was calculated from the amplification curves with LinReg v2013.0 [Ruijter et al., 2009; Tuomi et al., 2010].

**Table I:** Sequences of All Primer Pairs Used in the qRT‐PCR Experiments

Target	Amplicon size	5′→3′ sequence	Target	Amplicon size	5′→3′ sequence
*INSR*	260	AACATCCGAGGGGGCAACAA	*PPARγ*	142	GTGCCAGTTTCGATCCGTAGA
		TGGAAGAAGAGCTTGCCCTG			GGCCAGCATCGTGTAGATGA
*CAV1*	91	GCGACCCCAAGCATCTCAA	*aP2*	110	TTTGGTCACCATCCGGTCAG
		ATGCCGTCGAAACTGTGTGT			TGATGCTCTTCACCTTCCTGTC
*PTN*	73	ATGTCGTCCCAGCAATATCAGC	*C/EBPα*	129	GCCCCGTGAGAAAAATGAAGG
		CCAAGATGAAAATCAATGCCAGG			ATCCCCAACACCTAAGTCCC
*PI3K*	122	CGAGAGTGTCGTCACAGTGTC	*GLUT4*	157	TTTGCACACGGCTTCCGAAC
		TGTTCGCTTCCACAAACACAG			GGGTTCCCCATCGTCAGAGC
*AKT1*	116	ATGAACGACGTAGCCATTGTG	*ADIPOQ*	137	GACACCAAAAGGGCTCAGGA
		TTGTAGCCAATAAAGGTGCCAT			CACAAGTTCCCTTGGGTGGA
*GSK3β*	189	TGGCAGCAAGGTAACCACAG	*CPT1*	128	GCCCTGAGACAGACTCACAC
		CGGTTCTTAAATCGCTTGTCCTG			GTCCATTTTCCTTCCGTGCG
*HPRT*	136	GAGAGCGTTGGGCTTACCTC	*RAGE*	228	AGTCTACCAGATTCCTGGGA
		ATCGCTAATCACGACGCTGG			AGCTCTGACCGCAGTGTAAA

### FATTY ACID UPTAKE

Fully differentiated 3T3‐L1 adipocytes were seeded at a density of 1.97 × 10^5^ cells cm^−2^ in 96‐well plates and allowed to settle for 4 h after a brief centrifugation step (1000 rpm, 4 min). Subsequently, cells were starved with serum‐free growth medium for 1 h prior to incubation with CML (0.05–500 μM) or insulin (100 nM) for 30, 60, or 90 min at 37°C, and 5% CO_2_ in a humidified incubator. Free fatty acid uptake was then measured by adding loading buffer (100 μL, QBT fatty acid uptake kit, Molecular Devices) to each well and recording the emission at 515 nm every 20 s for a total of 60 min. The excitation wavelength was set to 485 nm following the manufacturer's instructions. The recorded emission was plotted versus time and the area below the curve used in data calculation.

### GLUCOSE UPTAKE

Fully differentiated 3T3‐L1 adipocytes were maintained in fresh growth medium (100 μL), with a serum concentration of 0.1% BSA for 4 h prior to the experiment. The starved cells were incubated with CML (50 μM) and 2‐deoxy‐d‐glucose (1 mM) for 5–120 min, subsequently washed twice with PBS, and once with ddH_2_O prior to the addition of ddH_2_O (100 μL) and freezing the samples. The samples were thawed, heated to 85°C for 45 min and triethanolamine solution (50 μL, 150 mM, pH 8.1) was added. Enzyme cocktail was prepared by mixing KCl (50 mM), diaphorase (1 U mL^−1^), MgCl_2_ (0.5 mM), BSA (0.02%), G6DPH (16 U mL^−1^), ATP (670 μM), NADP (0.12 μM), resazurin (25 μM), and triethanolamine (50 mM). In addition, a 2‐deoxy‐d‐glucose standard series (0–1000 ng) was prepared of which 50 μL were used in the assay for quantification purposes. Enzyme cocktail (150 μL) was added to each well and incubated at 37°C for 90 min prior to recording the fluorescence. The protein concentration (Protein kit, Pierce) of each sample was determined for normalization purposes.

### STATISTICAL ANALYSIS

Data are presented as average ± standard deviation. With the exception of the miRNA microarray experiment, all data were calculated from at least three independent biological replicates as stated in the legends of the corresponding figures and tables. Testing for significant differences between treatment and control was carried out with SigmaPlot 11.0 software. The statistical test carried out is stated in the legend of the corresponding table or figure, employing *t*‐tests in comparisons between treatment and control, one‐way ANOVA in comparisons between control and several concentrations of compound, and two‐way ANOVA in comparisons between control and treatment in cases of time‐dependency. Treatments with CML are shown in relation to untreated control denominated fold‐change (control = 1) or treated over control (T/C, control = 100%).

## RESULTS

### MTT ASSAY

In order to exclude negative effects of long‐term treatment with 0.5–500 μM CML on the metabolic activity of 3T3‐L1 cells, MTT assays were carried out in which cells were treated with 0.5–500 μM CML for a total of 12 days starting at differentiation, corresponding to the experimental setting of the Oil Red O staining. No statistically significant differences between untreated control and treated cells were detected (one‐way ANOVA, *P *> 0.05, data not shown).

### OIL RED O STAINING

**Figure 1:**
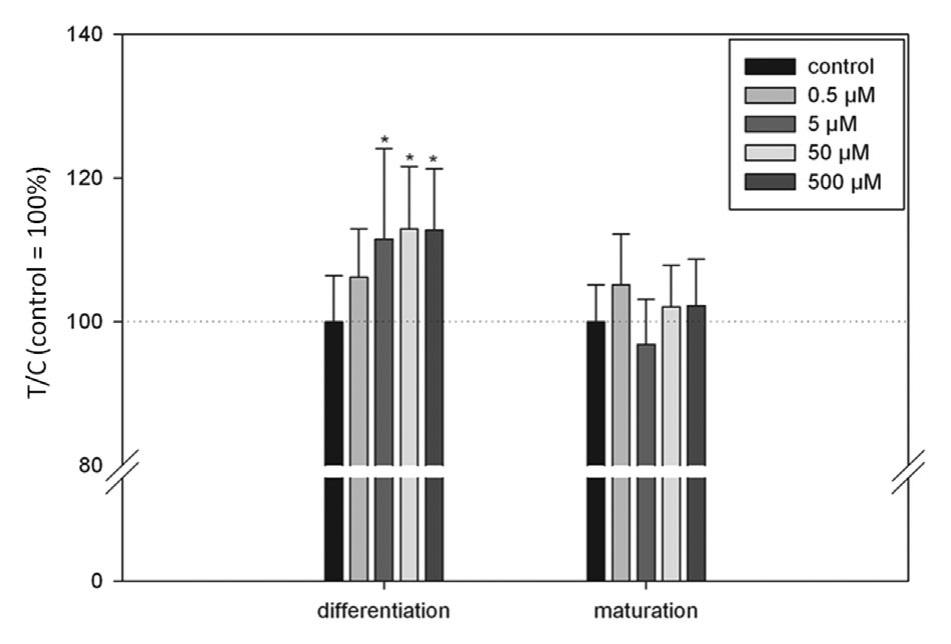
Results of lipid accumulation assessed by Oil Red O staining after starting CML treatment at differentiation or maturation. Data are displayed as average ± standard deviation in relation to untreated control (set to 100%), calculated from at least three biological replicates. Data were tested for outliers employing Nalimov outlier test. Statistics: one‐way ANOVA followed by Holm–Sidak post hoc test, overall significance level *P* = 0.01 versus control.

For the purpose of quantifying the degree of lipid droplet accumulation in the cytoplasm of cells, triglycerides, and cholesteryl oleate are specifically stained with the lysochrome diazo compound Oil Red O [Ramirez‐Zacarias et al., 1992]. To assess the influence of CML on lipid accumulation, 3T3‐L1 preadipocytes were treated with the compound, starting at differentiation, or at maturation. Cells in which the incubation with CML was initiated during differentiation accumulated more lipid droplets than untreated controls. In 3T3‐L1 cells treated with 500 μM CML lipid accumulation increased by 12.8 ± 8.5% (*P* = 0.002 vs. control, one‐way ANOVA followed by Holm–Sidak post hoc test) compared to untreated controls (Fig. 1). Similar results were obtained for 50 μM (12.9 ± 8.6%, *P* = 0.002 vs. control) and 5 μM (11.5 ± 12.6%, *P* = 0.004 vs. control). However, when CML was added for the first time during maturation, it did not impact the degree of droplet accumulation in any of the tested concentrations (0.5–500 μM, *P *> 0.05).

### miRNA EXPRESSION

**Figure 2:**
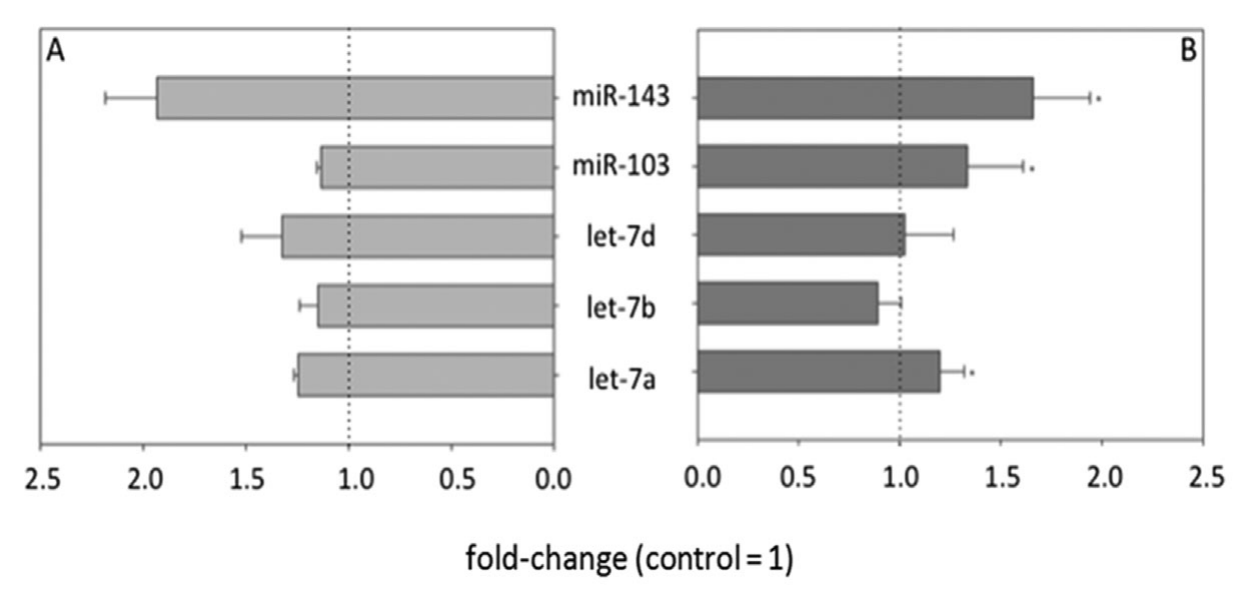
Comparison of results obtained by microarray (A) and ddPCR (B) for selected microRNAs. (A) Microarray results are displayed in relation to untreated control (set to 1), calculated from one biological sample, with several technical replicates being present on the chip, (B) ddPCR results are displayed in relation to untreated control (set to 1), calculated from at least four biological replicates, Statistics: *t*‐test versus control, * = *P* < 0.05.

**Figure 3:**
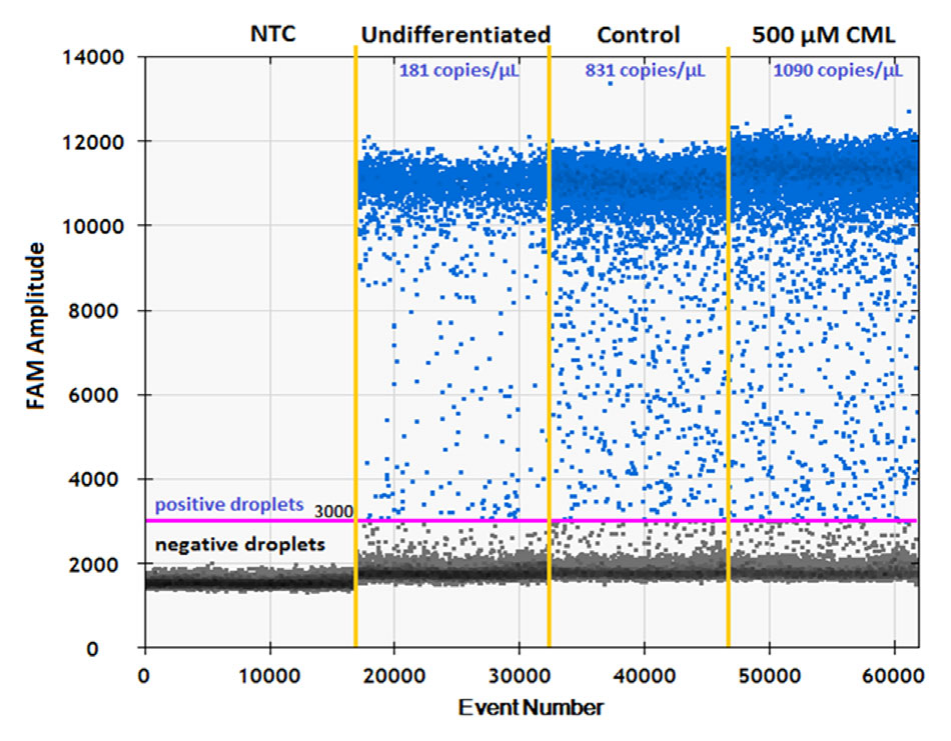
Example of ddPCR output showing negative (bottom, black) and positive (top, blue) droplets of negative control, undifferentiated cells, untreated control, and cells treated with 500 μM CML.

MicroRNAs are small non‐coding RNAs for which regulatory functions on mRNAs involved in adipogenesis have been proposed [McGregor and Choi, 2011; Peng et al., 2014]. In the present study, customized miRNA microarrays comprising probes for all mature murine miRNA sequences available in the Sanger miRNA database (Release 19) were used for identifying miRNAs influenced by treatment with 500 μM CML starting at differentiation. Figure 2 shows the fold‐change of selected miRNAs, chosen due to their association with the regulation of glucose homeostasis and adipogenesis, in relation to untreated controls for both miRNA microarray, and digital droplet PCR (ddPCR), showing good correlation between the two methods. The selected miRNAs were further investigated by ddPCR, which owes its high sensitivity to the capability of individually reading the fluorescence signal of 20,000 droplets, each with sample partitioned into it during droplet generation. This method allows for an absolute quantification of targeted miRNAs. The expression of mmu‐miR‐143‐3p (6.97 ± 5.38 copies μL^−1^) and mmu‐miR‐103‐1‐5p (14.59 ± 11.86 copies μL^−1^) was increased in mature adipocytes on the 9th day after induction of differentiation compared to undifferentiated 3T3‐L1 cells on day 0. When comparing untreated, fully differentiated control cells to cells that had been treated with 500 μM CML for 9 days, the treatment further up‐regulated the expression of miR‐103, miR‐143, and let‐7a. In relation to untreated mature adipocytes fold‐changes of 1.34 ± 0.28 (*P* < 0.001, *t*‐test vs. control), 1.66 ± 0.45 (*P* = 0.008, *t*‐test vs. control), and 1.20 ± 0.12 (*P* = 0.029, *t*‐test vs. control) were observed, respectively (Fig. 2). Figure 3 displays an example of the QuantaSoft output after a ddPCR run, clearly showing two populations of droplets, the low amplitude droplets being negative for the target and the high amplitude droplets being positive for the target, and thus, exhibiting high FAM (fluorescein) fluorescence.

### qRT‐PCR

**Figure 4:**
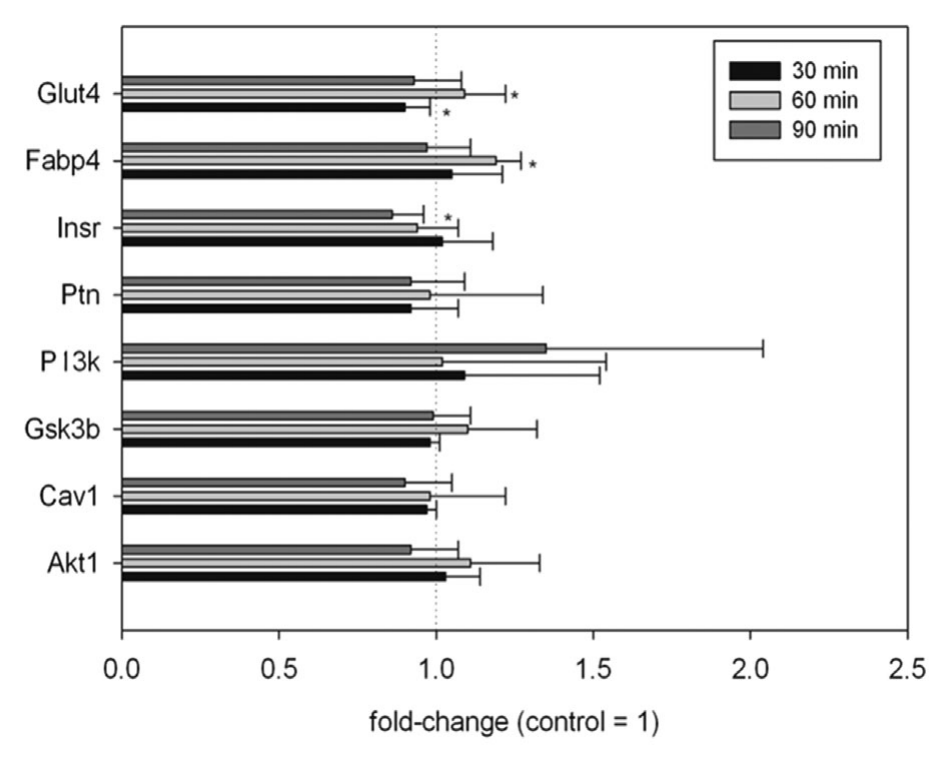
qRT‐PCR results of short‐term treatment of mature adipocytes with 500 μM CML for 30, 60, or 90 min. Data are displayed as average ± standard deviation in relation to untreated control (set to 1), calculated from at least three biological replicates. Statistics: two‐way ANOVA followed by Holm–Sidak post hoc test.

The influence of CML on adipogenesis and on fully differentiated adipocytes after treatment for 30, 60, or 90 min was assessed by qRT‐PCR. In both sets of experiments, potential targets of miR‐103, miR‐143, and let‐7 were analyzed. In addition, genes associated with adipogenesis were investigated in the experiments in which CML was added starting at the induction of differentiation 2 days post‐confluence. The long‐term treatment of differentiating 3T3‐L1 cells with 500 μM CML did not show any effects on *PPARγ*, *C/EBPα*, *INSR*, or *RAGE* (Table II). However, a regulation of *AKT1*, *PTN*, *CAV1*, and *PI3 K*, *CPT1α*, *Adipoq*, *GLUT4*, and *aP2* was observed (Table II). In contrast, no effect on *AKT1*, *PTN*, *CAV1*, and *PI3 K* was seen in the experiments in which fully differentiated 3T3‐L1 cells were treated with 500 μM CML for 30, 60, or 90 min (Fig. 4). In this case, effects on *GLUT4*, *INSR*, and *aP2* were observed. Glucose transporter type 4 was down‐regulated (0.90 ± 0.08, *P* < 0.05, two‐way ANOVA vs. control followed by Holm–Sidak post hoc test) after 30 min treatment with 500 μM CML. In addition, *INSR* also showed a down‐regulation (0.86 ± 0.10, *P *< 0.05 vs. control) after 90 min. Fatty acid binding protein 4, was slightly up‐regulated (1.19 ± 0.08, *P* < 0.05 vs. control) after 60 min.

**Table II:** qRT‐PCR Results of 3T3‐L1 Cells Treated With 500 μM CML During Differentiation

Target	Control	500 μM CML	*P*‐value
*PPARγ*	1.00 ± 0.05	0.97 ± 0.14	>0.05
*C/EBPα*	1.00 ± 0.04	0.96 ± 0.23	>0.05
*aP2*	1.00 ± 0.06	0.89 ± 0.09	<0.001
*CPT1*	1.00 ± 0.07	0.86 ± 0.15	0.016
*ADIPOQ*	1.00 ± 0.06	0.90 ± 0.19	0.009
*RAGE*	1.00 ± 0.08	1.15 ± 0.23	>0.05
*GLUT4*	1.00 ± 0.04	1.08 ± 0.10	0.007
*INSR*	1.00 ± 0.05	1.04 ± 0.18	>0.05
*CAV1*	1.00 ± 0.03	1.08 ± 0.18	0.043
*AKT1*	1.00 ± 0.08	1.32 ± 0.09	<0.001
*PTN*	1.00 ± 0.06	1.30 ± 0.16	<0.001
*PI3K*	1.00 ± 0.41	1.70 ± 0.69	0.009
*GSK3β*	1.00 ± 0.06	1.15 ± 0.14	0.003

Data are presented as average ± standard deviation in relation to untreated control (set to 1), calculated from at least four biological replicates, statistics: *t*‐test versus control.

### FATTY ACID UPTAKE AND GLUCOSE UPTAKE

**Figure 5:**
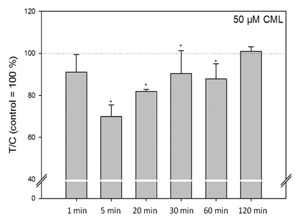
Results of glucose uptake experiments after treatment with 50 μM CML. Data are displayed as average ± standard deviation in relation to untreated control (set to 100%), calculated from at least three biological replicates. Statistics: two‐way ANOVA versus control followed by Holm–Sidak post hoc test, * = *P* < 0.05.

In order to determine whether CML changes the uptake of free fatty acids, fully differentiated 3T3‐L1 cells were treated with concentrations of 0.05–500 μM CML for 30, 60, or 90 min. However, no effects were observed at any of the tested time points (Table III). Similarly, the influence of 50 μM CML on glucose uptake was tested in fully differentiated 3T3‐L1 adipocytes. Treatment with 50 μM CML for 5 min down‐regulated glucose uptake (Fig. 5), leading to a value of 69.91 ± 5.63% (*P* < 0.05, two‐way ANOVA vs. control followed by Holm–Sidak post hoc test) in relation to untreated control. In addition, glucose uptake was reduced at 20, 30, and 60 min with values of 81.77 ± 1.13% (*P* < 0.05 vs. control), 90.48 ± 10.89% (*P* < 0.05 vs. control), and 87.89 ± 7.28% (*P* < 0.05 vs. control), respectively.

**Table III:** Fatty Acid Uptake After Treatment With 0.05–500 μM CML for 30, 60, or 90 min

	30 min	60 min	90 min
Control	100 ± 2.7	100 ± 4.6	100 ± 2.6
Insulin	142 ± 10.9*	140 ± 5.4*	137 ± 10.8*
0.05 μM	104 ± 6.6	94.1 ± 4.6	98.4 ± 5.1
0.5 μM	102 ± 8.4	96.9 ± 3.9	97.3 ± 14.2
5 μM	100 ± 8.5	111 ± 16.2	96.5 ± 11.6
50 μM	104 ± 8.8	104 ± 9.6	98.0 ± 11.6
500 μM	105 ± 8.9	98.3 ± 7.1	98.3 ± 6.0

Data are presented as average ± standard deviation in relation to untreated control (set to 100), calculated from at least four biological replicates, statistics: one‐way ANOVA versus control.

* *P *< 0.05.

## DISCUSSION

Among the group of AGEs, CML has been identified as major epitope of proteins carrying AGE modifications [Ikeda et al., 1996] and is frequently encountered in a Western diet. CML content varies in the diverse food categories ranging from up to 0.53 mg per average portion size in fruits and vegetables to 84.8 mg per average portion size in meat dishes [Hull et al., 2012]. A study by Hellwig et al. [2014] on CML absorption availability after simulated gastrointestinal digestion of casein samples reported liberation of CML comparable to that of native amino acids, concluding that CML present should be available for absorption after release from peptides below 1,000 Da in size. While most studies investigated the effects of AGEs, including CML, protein bound to either albumin or casein, a number of reports on the influence of free CML are available. Free CML has been detected in urine and plasma samples of healthy, and compromised individuals [Friess et al., 2003]. A determination of the urinary excretion of protein‐bound and free CML showed approximately 60% of the total excreted CML to fall within the protein‐ and peptide‐bound fraction, thus, leaving up to 40% as free CML [Friess et al., 2003]. Similarly, a study on 1291 participants classed into groups of normal glucose metabolism, impaired glucose metabolism, and manifested type 2 diabetes, reported up to 100 nM free CML in the analysed plasma samples [Hanssen et al., 2013]. These reports indicate that CML may not only be of interest in protein modifications, but also if used in experiments in its free form. In the present study, we aimed at investigating the effects of protein‐free, exogenous CML on murine 3T3‐L1 adipocytes. Firstly, 500 μM CML were added to the cell culture medium starting at differentiation. Secondly, preadipocytes were differentiated under standard conditions to mature 3T3‐L1 adipocytes and incubated with 0.05–500 μM CML, representing low concentrations close to the plasma concentration of free CML, moderate concentrations corresponding to plasma levels of protein‐bound CML, and high CML concentrations which may be achieved locally by accumulation.

In the first set of experiments, in which 500 μM CML were added starting at differentiation, all murine miRNAs recorded in the Sanger database were analyzed by means of customized microarrays. A subset of the miRNAs, found to be regulated by CML on the microarray, was selected and quantified using digital droplet PCR. This subset of miRNAs consisted of mmu‐miR‐103‐1‐5p, mmu‐miR‐143‐3p, let‐7a, let‐7b, and let‐7d. Within this group of miRNAs, expression of miR‐143, miR‐103, and let‐7a was up‐regulated by CML. In recent years, miRNAs have been described as regulators of AGE/RAGE signaling and have been discussed as potential therapeutic targets in the management of diabetic complications [Piperi et al., 2015]. Both miR‐143 and miR‐103 have been described as regulators of glucose homeostasis. Trajkovski et al. [2011] demonstrated an up‐regulation of miR‐103 and miR‐107 in obese mice. In addition, the authors showed gain of miR‐103/107 function in liver or fat to result in impaired glucose homeostasis, and identified caveolin‐1 (CAV‐1) as target of these two miRNAs [Trajkovski et al., 2011]. Similarly, conditional over‐expression of miR‐143 in mice has been shown to impair glucose metabolism and insulin‐stimulated AKT activation in the liver of these animals [Jordan et al., 2011]. Based on these results, we next analyzed the expression of genes involved in the AKT pathway, as well as the miR‐103 target *CAV‐1*, by qRT‐PCR. In addition, we analyzed the expression of *PPARγ*, a key regulator in adipogenesis and target of the let‐7 group, as we observed an up‐regulation of let‐7a by CML. Our qRT‐PCR results indicated a regulation of *PTN*, *PI3K*, *AKT1*, *GSK3β*, *GLUT4*, and *CAV‐1* by CML, but not of the key transcription factors *PPARγ* or *C/EBPα*. A study by Yang et al. [2013] revealed treatment with 100 μg/mL AGEs to enhance adipogenesis in 3T3‐L1 cells as increased levels of lipid accumulation, assessed by Oil Red O staining 12 days after induction of differentiation, and higher protein levels of PPARγ, C/EBPα, and aP2 5 days after induction of differentiation were observed. Furthermore, Yang et al. [2013] showed BSA‐derived AGEs dose‐dependently activate Akt, an effect which was attenuated in siRNA experiments lowering CAV‐1 levels, by abolishing caveolae using β‐methylcyclodextrin and treatment with a PI3‐kinase inhibitor. Thus, Yang et al. [2013] suggested the pro‐adipogenic effects of AGEs to be related to AKT, PI3‐kinase, and IGF‐1 receptor. Here, we show regulation of *AKT*, *CAV‐1*, and *PI3K* by free CML on a gene level.

Ectopic let‐7a expression has been associated with decreased PPARγ levels and inhibition of differentiation of 3T3‐L1 cells [Sun et al., 2009]. However, the let‐7 group has also been described in the context of insulin sensitivity and glucose homeostasis. Frost and Olson [2011] reported global over‐expression of let‐7 to impair glucose tolerance and reduce fat mass, while knockdown resulted in improvements in insulin sensitivity in muscle and liver of C57BL/6 mice. Also, miRNAs miR‐27, miR‐130, miR‐378/378*, and Hzf have been reported to target *PPARγ* and *C/EBPα* which were not analyzed in this study (see review: [Kim and Kyung Lee, 2012]). Furthermore, we cannot exclude PPARγ regulation by CML earlier in adipogenesis, as we analyzed gene and miRNA expression only on day 9 of differentiation, on which cells were considered mature adipocytes and previous reports of AGEs on adipogenesis reported increased protein levels on day 5 of differentiation [Yang et al., 2013].

**Figure 6:**
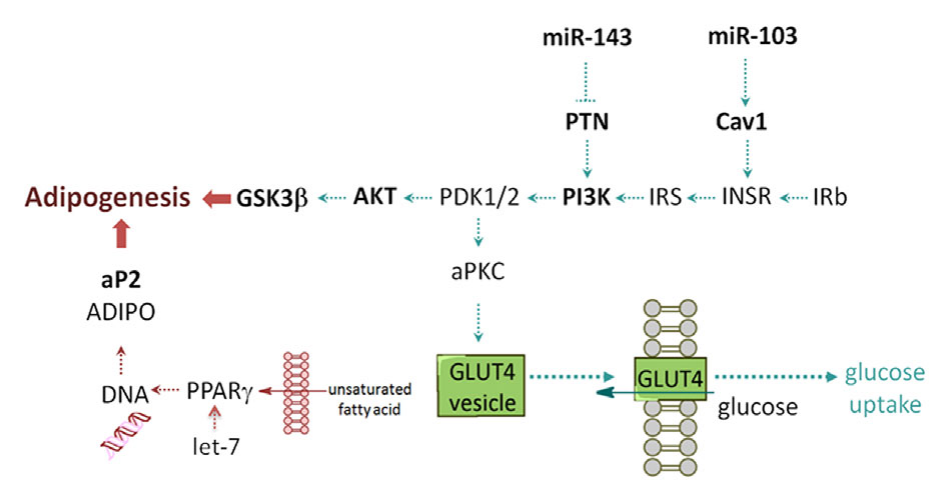
Graphical overview of some of the genes involved in adipogenesis and the miRNAs analyzed within this study. Targets regulated by CML are shown in bold.

In order to determine the effects of CML addition during differentiation on a functional level, we employed Oil Red O staining. In 3T3‐L1 cells treated with CML starting at differentiation, we observed an increase in lipid accumulation to similar extent after treatment with 5, 50, or 500 μM CML. This demonstrates CML to influence lipid accumulation also in plasma relevant concentrations, as plasma concentrations of healthy individuals centre around 2.8 μM [Teerlink et al., 2004], reaching around 12 μM in diabetic subjects with compromised renal function [Lieuw et al., 2004]. Furthermore, trapping of CML in adipose tissue has been reported [Jia et al., 2012], which may yield in even higher local concentrations. A higher degree of triglyceride accumulation, up‐regulation of associated markers early in adipogenesis, and hence, an accelerated adipogenesis has been shown in preadipocytes ectopically expressing miR‐103 and miR‐143 [Xie et al., 2009], two miRNAs differentially expressed after CML treatment in the present study. Also, we observed down‐regulation of the lipolytic CPT1 and the adipokine ADIPOQ, the expression of which has been described in the modulation of insulin sensitivity in 3T3‐L1 cells [Fasshauer et al., 2002], after CML treatment. Interestingly, we did not observe an increase in lipid accumulation when CML treatment was only begun at the maturation stage, that is, 2 days after induction of differentiation. This suggests CML may have a more pronounced impact on markers in the early stage of adipogenesis. Figure 6 shows a schematic overview of some of the genes we analyzed after long‐term treatment with CML during adipogenesis. In the experiments, in which preadipocytes were differentiated under standard conditions to mature adipocytes, CML showed no influence on free fatty acid uptake in any of the concentrations tested. We did, however, observe decreases in basal glucose uptake after 5, 20, 30, or 60 min treatment with 50 μM CML. A study by Unoki et al. [2007] reported AGE‐BSA, similarly to our findings, to prevent basal glucose uptake in 3T3‐L1 cells but, in addition, showed decreased insulin‐stimulated glucose uptake, from which the authors concluded an insulin‐independent interference of AGE‐RAGE with glucose uptake. Further, the authors reported that in KK‐A^y^ mice onset of insulin sensitivity impairment was linked to accumulation of AGE‐BSA in the adipose tissue of the animals. RAGE expression has been shown to be closely related to insulin sensitivity and adipocyte hypertrophy in mice [Monden et al., 2013]. A study by Monden et al. [2013] demonstrated RAGE over‐expression to result in decreased adiponectin and *GLUT4* expression, in addition to attenuating insulin‐stimulated glucose uptake. In RAGE‐knockout mice, less epididymal adipocyte size and fat weight was observed in addition to exhibiting higher insulin sensitivity compared to wild type mice [Monden et al., 2013].

In terms of gene expression, we did not note changes in *RAGE* expression after treatment with CML at any of the tested time points. We did, however, observe a down‐regulation of *INSR* after 90 min and a differential regulation of the insulin‐regulated glucose transporter 4 (*GLUT4*) after 30 and 60 min of treatment with 500 μM CML, indicating an impact of CML treatment on glucose homeostasis on a genetic level. CML has been identified as the major epitope of AGE‐modified proteins and activation of RAGE leading to a modulation of gene expression has been shown [Ikeda et al., 1996; Kislinger et al., 1999], yet, free CML has been demonstrated to not stably bind to RAGE [Xie et al., 2008]. However, a study on HEK‐293 cells expressing full length RAGE and HEK‐293 cells expressing a C‐terminally truncated version of the receptor showed casein‐CML, as well as free CML to increase p38 MAP kinase activation [Zill et al., 2003]. While an increase was still observed in cells lacking the cytosolic domain of RAGE, it was considerably reduced, suggesting a RAGE‐mediated pathway for both free and protein‐bound CML [Zill et al., 2003]. Similarly, a study by Schmid et al. [2008] reported increased expression of several heat shock proteins after treatment of Caco‐2 cells with casein‐CML and free CML, hypothesising RAGE‐involvement. In our previous experiments, on the human neuronal cell line SH‐SY5Y we observed changes in *RAGE* gene expression after incubation with 500 μM CML [Holik et al., 2013]. Our results are in accordance with previously published data on AGEs and adipogenesis as well as glucose uptake, however, the involvement of RAGE in effects seen after treatment with free CML, and the cellular fate of the administered CML should be further investigated. Furthermore, mechanistic studies into the changes in mRNA levels of members of the PI3K/AKT pathway should be conducted, including the investigation of possible alternations in protein or phosphorylation level. In summary, our findings show addition of free CML during differentiation of 3T3‐L1 preadipocytes to result in increased lipid accumulation. Furthermore, CML was shown to influence the expression of miRNAs associated with impaired glucose homeostasis and adipogenesis, as well as associated genes.
